# Role of rh-IGF-1/IGFBP-3 in the postnatal kidney maturation of preterm pigs: Not so simple

**DOI:** 10.1038/s41390-024-03426-7

**Published:** 2024-08-12

**Authors:** Patricio E. Ray

**Affiliations:** Department of Pediatrics and Child Health Research Center, University of Virginia School of Medicine, Charlottesville, VA USA

The Insulin-Like Growth Factor system (IGFs) comprised by IGF-1 and the IGF-2 ligands, IGF-receptors (IGF-R) and several IGF binding proteins (IGFBP) is considered essential for regulating fetal and postnatal growth, the action of growth hormone, as well as the growth of intestinal, lung, bone, brain and kidney, the maintenance of the extracellular fluid (ECF) volume, the regulation of the immune system, glucose metabolism, and other key physiological functions.^1,2^ In preterm infants, low levels of IGF-1 have been associated with the development of retinopathy of prematurity, bronchopulmonary dysplasia, necrotizing enterocolitis (NEC), intraventricular hemorrhages, and impaired neurodevelopment.^3^ Therefore, clinical trials have been done in preterm infants to determine whether injections of recombinant human (rh)-IGF-1 combined with rh-IGFBP-3, which prolongs IGF-1’s half-life and regulates its tissue accessibility and activity, could ameliorate these complications.^3^ This issue of *Pediatric Research* introduces a study by Zhong et al. showing that hr-IGF-1/IGFBP-3 given to preterm pigs delivered at 90% gestational age, promotes kidney maturation and mitigates the impact of preterm weight on kidney structure.^4^ Based on these findings, the authors suggested that hr-IGF-1/IGFPB-3 could sever as a promising therapy for kidney immaturity in preterm infants. Here, we argue that the interpretation of these data is not so simple, and will discuss relevant issues that need to be considered to assess the clinical relevance of these findings for preterm infants,

Preterm infants small for gestational age (SGA), intrauterine growth restriction (IUGR), and/or very premature (<32 weeks GA) are born with a reduced number of nephrons, given that nephrogenesis is completed in humans at ~ 36 weeks of GA, although some compensatory nephrogenesis occurs within the first days of neonatal life.^5^ According to Brenner’s glomerular hyperfiltration hypothesis,^6^ preterm infants born with a low number of nephrons are at risk of developing hypertension and chronic kidney diseases (CKD) later in life. Glomerular hyperfiltration is defined by a physiological increase in single nephron glomerular filtration rate (GFR) that takes place due to changes in glomerular capillary volume and pressure leading to the hypertrophy of glomeruli and proximal tubules. It is either a physiological or an abnormal response caused by abnormal afferent arteriolar vasodilatation or efferent arteriole vasoconstriction causing glomerular hypertension. In kidneys endowed with fewer or immature nephrons, this compensatory hypertrophy can increase the glomerular size as well as the reabsorption of sodium, leading to the expansion of the ECF volume, development of hypertension and secondary focal segmental glomerulosclerosis,^6^ at least in people with a genetic or acquired susceptibility to develop hypertension or renal disease. Furthermore, preterm infants are more likely to develop acute hypoxic or ischemia events, sepsis, and nephrotoxic insults, which acting as second hits can further decrease the number of functional nephrons.^5^ Thus, new approaches are needed to protect the kidney and renal function of preterm infants.

Given the potential adverse effects of glomerular hyperfiltration, experimental therapies based on renal growth factors, including IGF-1,^1,2^ should consider whether the desired effects can be accomplished without triggering a maladaptive hypertrophic response that in the long term can further worsen the kidney function. This goal is difficult to achieve, since we do not have effective clinical tools to determine the number of nephrons or identify glomeruli that are undergoing maladaptive hyperfiltration changes. Higher GFR values, microalbuminuria, and increased kidney size provide clues to identify infants at risk. However, given the variability in kidney size and glomerular number between individual preterm infants, and considering that eGFR formulas during the first weeks of life of preterm infants are inaccurate given that the serum creatinine (SCr) levels are not in a steady,^7^ these surrogate markers of hyperfiltration are not very sensitive or reliable. The study of Zhong et al. highlights this point, since they were unable to detect significant differences in eGFR, albuminuria, SCr and kidney size between the preterm pigs treated or not with hr-IGF-1/hr-IGFBP-3 despite the remarkable kidney histological changes noted.^4^ Furthermore, surprisingly, the control term pigs showed higher SCr and BUN levels and higher urine albumin/Cr ratios on day nine postnatally, compared to the preterm groups. The latter findings, remain unexplained and are in disagreement with the studies done in preterm infants.^7^ It is also well known that injections of IGF-1 in humans increase their renal plasma flow and GFR, and that IGF-1 increases the single nephron GFR in rats by increasing the ultrafiltration coefficient and decreasing arteriolar resistance through nitric oxide and prostaglandins mediated mechanisms.^1,2^ IGF-1 also increases the reabsorption of sodium and phosphate and expands the ECF volume.^1,2^ All these events may increase the blood pressure and induce glomerular hyperfiltration, placing nephrons at higher risk of premature failure. Unfortunately, Zhong et al., did not explore or discuss how hr-IGF-1/IGFBP-3 affected the blood pressure or the glomerular hyperfiltration process in their preterm pig model.

On the other hand, the kidney lesions noted by Zhong et al. in the control preterm pigs at post-natal day 5 (40% glomerular cystic dilatation, 31.8% glomerular hemorrhages, 50% tubular dilatation; 31% proximal tubule vacuolization; 36.4% interstitial edema and hemorrhages), were much more severe than those expected or seen in premature infants not subjected to additional kidney insults.^5^ The authors stated that these lesions resemble those seen in a cecal ligation and puncture rat model. It is worth mentioning that Zhong et al. used an experimental model system originally designed to define how hr-IGF1-IGFBP-3 affected the development of NEC in pre-term pigs.^4^ In their model, NEC develops spontaneously due to the preterm delivery by cesarean section plus the formula feeding, following the occurrence of hypothermia and hypoxia in the immediate neonatal period. Their previous studies showed that these events induced a significant inflammatory intestinal and systemic response, and therefore, the possibility that hr-IGF-1/IGFBP-3 might have prevented the kidney damage by minimizing this inflammatory response cannot be excluded. Zhong et al. claim that they selected pigs with minor or no intestinal lesions, but they also recognized that pigs with small intestinal lesions showed enlarged kidneys, and that on postnatal day 5 the preterm IGF-1 group showed a lower incidence of NEC compared to the preterm control group. Furthermore, pigs unlike humans, are programmed to complete nephrogenesis three weeks after birth, and they show many other differences related to the nephrogenesis process,^8^ the glomerular perfusion and compensatory kidney enlargement mechanism (e. g. increasing the number of nephrons rather that nephron hypertrophy),^9^ the rapid postnatal growth rate,^10^ and other physiological renal functions.^5,10^ When all these factors are taken into consideration, the assumption that a 90% GA preterm pig kidney (~70% completed nephrogenesis) equals a 70% preterm human kidney is questionable at best, at least in this model. Considering that the cesarean section, the artificial nurture and the weaning processes all combined were such a stressful event for the preterm pigs, we speculate that the immature kidney vasculature of pre-term pigs might be less likely to tolerate the changes required to establish the renal blood flow and glomerular pressure at birth, when compared to preterm infants. This possibility may explain why the pre-term pigs developed multiple glomerular and interstitial hemorrhages which appear to improve spontaneously over time. In this scenario, hr-IGF1/IGFPB-3 could act by minimizing the endothelial dysfunction and renal tubular damage as described in previous rodent models of AKI.^1,2^ However, an specific role of IGF-1 in renal glomerular angiogenesis or preventing glomerular neonatal hemorrhages has not been described before, and more studies are needed to determine whether IGF-1 can indeed play such a role, and if so, what is the mechanism.

In summary, there is a need to understand in depth the processes of nephrogenesis and kidney maturation in preterm pigs in comparison to humans and to develop relevant preterm animal models to test new renal therapies. Recent studies showing that genetically engineered pig kidneys could be transplanted successfully and function in humans will stimulate future research in a significant manner.^10^ For example, the pig growth hormone receptor gene is one of the candidate genes “to be deleted” prior to the transplantation of pig kidneys into humans to avoid its excessive growth in humans.^10^ On the other hand, clinical trials in which hr-IGF-1/IGFBP-3 was injected into preterm infants concluded that this treatment is safe.^3^ However, it is unclear whether these studies assessed the effects of hr-IGF-1/IGFB3 in the preterm kidney or whether the neonates were followed for a long enough time to exclude potential renal or hypertensive adverse effects. Previous human clinical trials in which IGF-1 or growth hormone were injected into critically ill patients to treat AKI or improve their clinical outcome, either failed or showed that growth hormone increased the mortality of critically ill adult patients.^1,2^ Hopefully, the study by Zhong et al. will stimulate future research to develop the best possible animal model system to test new kidney protective therapies for preterm infants, and to understand how the IGF-1 system affects the process of kidney maturation, growth and renal function of preterm pigs and infants.
